# Single-cell dissection of a collision marrow with coexisting acute myeloid leukemia and a Waldenström-related lymphoplasmacytic compartment

**DOI:** 10.3389/fonc.2026.1844537

**Published:** 2026-09-11

**Authors:** Xin Cao, Wei Lu, Hongming Huang

**Affiliations:** 1 Department of Hematology, Affiliated Hospital of Nantong University, Nantong, Jiangsu, China; 2 Department of Special Examination Center, Affiliated Hospital of Nantong University, Nantong, Jiangsu, China

**Keywords:** acute myeloid leukemia, collision marrow, CopyKAT, immune microenvironment, single-cell RNA sequencing, waldenström macroglobulinemia

## Abstract

**Background:**

The concurrent presence of acute myeloid leukemia (AML) and a Waldenström macroglobulinemia (WM)-related compartment within the same bone marrow is uncommon. Conventional diagnostics can establish coexistence but cannot completely resolve the cellular architecture and shared immune context of collision marrows.

**Methods:**

We performed single-cell RNA sequencing on the diagnostic bone marrow aspirate from a 67-year-old man with AML showing myelomonocytic features and a flow-validated WM-related B/plasma-cell compartment. Transcriptomic findings were integrated with marrow flow cytometry, serum studies, orthogonal MYD88 L265P testing, and longitudinal clinical assessment. Descriptive analyses included unsupervised clustering, lineage annotation, copy-number inference, AML state scoring, T-cell/natural killer (T/NK) subclustering, and exploratory cell-cell communication inference.

**Results:**

Single-cell profiling resolved AML-related myeloid, WM-related B/plasma-cell, T/NK, monocyte, and erythroid/megakaryocytic compartments. Copy-number variation (CNV) inference localized the dominant CNV-bearing population to AML-associated regions, with AML cells distributed across immature/progenitor-like, granulocytic-primed, and inflammatory/myelomonocytic-like states. The WM-related compartment comprised coexisting B-cell-like and plasma-cell-like states, with limited CNV resolution by transcriptome-based inference. Flow cytometry, monoclonal IgM, and *MYD88* L265P supported a clonal WM-related process. Immune profiling identified an effector-dominant T/NK milieu with a discrete *KLRC1*-high cytotoxic subset and a T/NK-centered inhibitory signaling landscape. After azacitidine plus venetoclax, both compartments initially decreased but later showed dissociated kinetics with AML re-expansion, persistent low-level WM-related marrow involvement, and sustained serologic abnormality.

**Conclusions:**

Integrated single-cell and orthogonal profiling showed that this collision marrow comprised an AML-dominant CNV-bearing leukemic compartment and a clinically anchored WM-related lymphoplasmacytic compartment, with dissociated kinetics under the same regimen. These findings illustrate the exploratory value of compartment-aware single-cell analysis in composite marrow malignancies.

## 1 Introduction

Concurrent involvement of the same bone marrow by a myeloid malignancy and a lymphoplasmacytic neoplasm is uncommon, and the biologic basis of such collision marrows remains incompletely understood. Acute myeloid leukemia (AML) is a heterogeneous malignancy characterized by multiple developmental states, whereas Waldenström macroglobulinemia (WM) is a mature B-cell neoplasm characterized by marrow infiltration by clonal lymphoplasmacytic cells and monoclonal IgM; *MYD88* mutations are common but are not required for diagnosis (1). Historical literature and rare contemporary case reports indicate that acute leukemia and macroglobulinemia can coexist, but such presentations remain distinctly uncommon (2–4). Although routine morphology and flow cytometry are well suited to establishing coexistence, they are less capable of resolving clonal architecture, lineage organization, and shared microenvironmental context.

Single-cell transcriptomic profiling is well suited to such complex marrow presentations because it can simultaneously resolve malignant cell states, state organization, and immune contexture (5). In AML, this approach has been instrumental in defining leukemic hierarchies, including immature, progenitor-like, granulocytic, and monocytic programs relevant to disease progression and host immunity (6). Emerging single-cell studies in WM likewise support tumor-cell heterogeneity and an immunosuppressive marrow microenvironment (7–10). These observations suggest that single-cell analysis may be particularly informative in collision marrows containing both AML and a WM-related component, where the central unresolved question is not simply whether both entities are present, but how they are organized and how they coexist within a shared marrow niche.

Here, we applied single-cell RNA sequencing to the diagnostic bone marrow of a patient with concurrent AML and a flow-validated, kappa-restricted WM-related B/plasma-cell compartment, integrating single-cell profiling with marrow flow cytometry, serologic studies, and longitudinal clinical assessment. Lineage identity was established by conventional morphology and flow cytometry; single-cell profiling was not used to diagnose either lineage, to replace routine diagnostics, or to infer resistance mechanisms from a baseline sample. Instead, we addressed three descriptive questions: whether transcriptome-derived copy-number variation (CNV) inference and reference-guided scoring could resolve disease-related state architecture beyond lineage assignment; which transcriptional programs were compartment-biased and which were broadly shared across the collision marrow; and which baseline immune- and niche-associated hypotheses could be nominated for future validation. Longitudinal clinical data were used only to describe compartment-specific kinetics, not to infer a mechanism of resistance.

## 2 Methods

### 2.1 Patient and clinical evaluation

A 67-year-old man presented with fatigue and had an ECOG performance status of 2. No relevant occupational, chemical, or radiation exposures or family history of hematologic malignancy were documented. He was evaluated at the Affiliated Hospital of Nantong University for AML with myelomonocytic features and a concomitant IgM-kappa clonal B/plasma-cell compartment. Initial diagnostic assessment included peripheral blood and marrow morphology, multiparameter flow cytometry, serum immunoglobulin quantification, immunofixation electrophoresis, and orthogonal *MYD88* mutation testing. The bone marrow biopsy report documented AML and a lymphoplasmacytic component but did not quantify lymphoplasmacytic infiltration. No definite multilineage dysplasia was identified on the available aspirate smear, and comprehensive myeloid next-generation sequencing was not performed. Single-cell RNA sequencing was performed on the diagnostic bone marrow aspirate. The patient subsequently received azacitidine plus venetoclax, with follow-up marrow examinations used to describe both compartments. This study was approved by the institutional review board of the Affiliated Hospital of Nantong University (approval number 2017-L045) and was conducted in accordance with the Declaration of Helsinki. Written informed consent was obtained from the patient.

### 2.2 MYD88 mutation testing

Orthogonal *MYD88* L265P testing was performed on a CD19-sorted B-cell fraction obtained from the diagnostic bone marrow aspirate. Briefly, bone marrow mononuclear cells were prepared using standard clinical laboratory procedures, after which CD19-positive B-cell enrichment was performed using CD19 MicroBeads, human (Miltenyi Biotec, Bergisch Gladbach, Germany; catalog no. 130-050-301) according to the manufacturer’s instructions. Post-enrichment purity was not prospectively recorded; therefore, the result was not interpreted as a single-cell or quantitatively clonal measurement. The canonical *MYD88* c.794T>C (p.L265P) hotspot variant was interrogated by allele-specific polymerase chain reaction (AS-PCR) according to the workflow used by the testing laboratory. A positive *MYD88* L265P result was obtained. Because this assay was performed on an enriched CD19-positive fraction rather than at single-cell resolution, the result was interpreted as orthogonal molecular support for a WM-related clonal B-cell process in conjunction with marrow flow cytometry, serum immunofixation findings, and the single-cell-defined B/plasma-cell state spectrum.

### 2.3 Single-cell RNA sequencing and preprocessing

Bone marrow mononuclear cells were isolated from diagnostic bone marrow aspirates by density-gradient centrifugation. Single-cell libraries were generated using the Chromium Single Cell 3′ Reagent Kit (10x Genomics, Pleasanton, CA, USA) according to the manufacturer’s protocol. Briefly, single cells were captured and barcoded using the Chromium Controller, followed by reverse transcription and complementary DNA (cDNA) library preparation. Libraries were sequenced on an Illumina NovaSeq 6000 platform to generate paired-end reads.

Raw sequencing data were processed using Cell Ranger (version 6.1.2, 10x Genomics) for sample demultiplexing, alignment to the GRCh38 human reference genome, barcode processing, and unique molecular identifier (UMI) counting. Downstream analyses were performed in R (version 4.5.2) using Seurat (version 5.4.0). Cells with nFeature_RNA <200 or >5000, or with a mitochondrial transcript proportion >10% were excluded, and genes expressed in fewer than three cells were removed before downstream analyses.

### 2.4 Cell annotation and B/plasma-state analysis

Normalized data were scaled and subjected to principal component analysis, followed by graph-based clustering and uniform manifold approximation and projection (UMAP) for visualization. Cell clusters were annotated according to canonical lineage markers together with cluster-level transcriptional features. Major compartments were defined as AML-related myeloid cells, WM-related B/plasma cells, T cells and natural killer cells (T/NK cells), microenvironment monocytes, and erythroid/megakaryocytic populations.

Representative markers used for annotation included *CD34, KIT, SPINK2, MPO*, and *LYZ* for AML-related myeloid populations; *CD19, MS4A1, CD79A, IGHM, JCHAIN, MZB1, XBP1*, and *SDC1* for the WM-related B/plasma-cell compartment; and *CD3D, IL7R, CD8A*, and *NKG7* for T/NK cells.

To further examine the internal structure of the WM-related compartment, these cells were subsetted and re-embedded in a separate UMAP space. Cells were visualized according to transcriptionally defined B-cell-like and plasma-cell-like states. Representative markers used to interpret the B-cell-like region included *CD19, MS4A1*, and *CD79A*, whereas *IRF4, XBP1, MZB1*, *SDC1*, and *JCHAIN* were used to interpret the plasma-cell-like region. The analysis did not test the anatomical origin or directional differentiation of either state.

### 2.5 CopyKAT-based CNV inference

To infer large-scale chromosomal copy-number abnormalities from transcriptomic data, we applied CopyKAT (1.1.0) to the filtered single-cell expression matrix using the recommended settings unless otherwise specified (11). Diploid reference cells were identified by the algorithm under its recommended settings rather than being manually prespecified. Cells were classified as aneuploid, diploid, or undefined. CopyKAT assignments were first projected onto the global UMAP to identify major CNV-bearing populations and were then summarized across annotated compartments.

Because transcriptome-derived CNV inference may be less stable in immune and plasmacytic populations, CopyKAT results for the WM-related compartment were interpreted conservatively and in the context of flow-cytometric findings, transcriptional state organization, and lineage-marker expression.

### 2.6 AML state scoring using a reduced Van Galen framework

AML-associated cells were subset for focused analysis of leukemic state structure. State-associated marker expression was examined using feature plots and lineage-informed dot plots. To provide an external reference for AML state organization, module scoring was performed using a reduced Van Galen framework comprising HSC-like, progenitor-like, GMP-like, promonocyte-like, monocytic-like, and cDC-like signatures (6). Module scores were visualized across AML-associated populations to assess their consistency with the observed myeloid hierarchy. A reduced-signature approach was used to preserve interpretability in a single-case dataset.

To describe baseline apoptotic-regulator heterogeneity, expression of *BCL2*, *MCL1*, *BCL2L1*, and *BCL2A1* was summarized across diagnostic AML transcriptional states using dot plots. No post-treatment sample underwent single-cell RNA sequencing; this analysis was not designed to predict venetoclax response or resistance.

### 2.7 Compartment-biased expression and reference-guided signature scoring

Compartment-biased and broadly expressed transcriptional programs were examined across major compartments using dot plots of log-normalized expression. Selected genes were grouped into myeloid/inflammatory, B/plasma-cell, and broadly expressed immune/niche-associated programs using literature-supported lineage or immune annotations. These displays were descriptive and were not used to define collision-specific biomarkers.

### 2.8 Visualization and network analysis

UMAPs, state maps, and compartment-level summaries were generated using ggplot2-based workflows in R. In dot plots, dot size represents the fraction of cells expressing each marker, and color intensity represents scaled average expression. Monocyte subclusters were visualized using marker-based dot plots.

To generate hypotheses about compartment-associated microenvironmental signaling, cell-cell communication analysis was performed using CellChat (version 1.6.1) (12). Inferred signaling patterns were summarized globally and further examined for T/NK-targeted communication and selected pathways, including MHC-I- and GALECTIN-related signaling. Ligand-receptor inference from single-cell transcriptomes is probabilistic and method-dependent (13); therefore, these analyses were considered supportive and hypothesis-generating. No inferred ligand-receptor interaction was validated at the protein or functional level by flow cytometry, spatial assays, or perturbation experiments.

### 2.9 Statistical analysis and scope of inference

This was a single-case study based on one pretreatment diagnostic single-cell RNA sequencing specimen. No patient-level inferential hypothesis testing was performed. Cell counts, percentages, expression summaries, and module-score distributions are reported descriptively. Individual cells were not treated as independent biological replicates. No *p*-values or confidence intervals were reported. Terms such as higher or lower refer only to within-sample descriptive differences and do not establish generalizable effects, treatment-response associations, or causal mechanisms.

## 3 Results

### 3.1 Baseline clinicopathologic features and a single-cell atlas of the collision marrow

**Figure 1:**
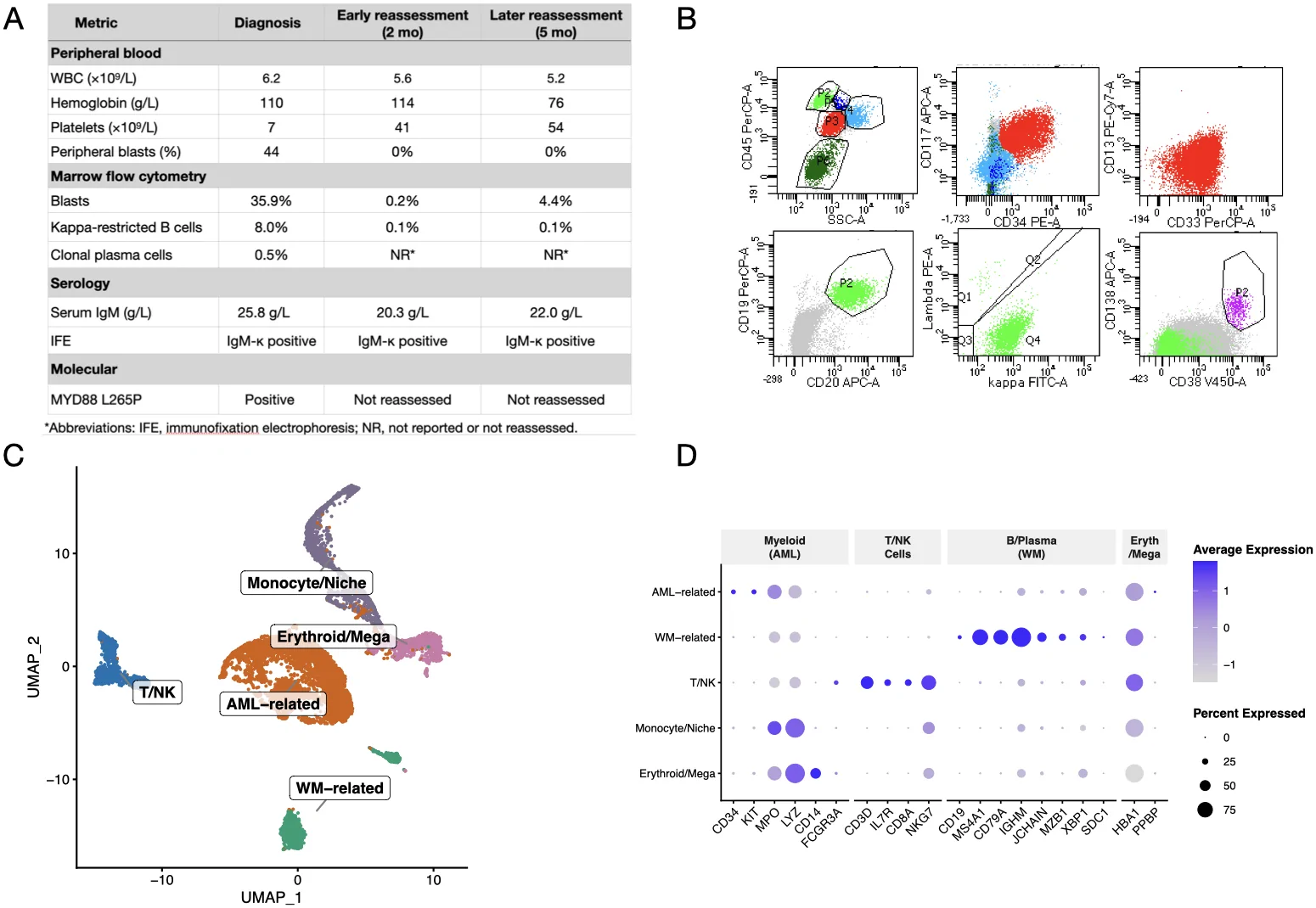
Baseline clinicopathologic findings and single-cell resolution of a collision marrow. **(A)** Baseline and longitudinal clinical summary integrating peripheral blood indices, marrow flow-cytometric findings, serum studies, and orthogonal *MYD88* L265P testing. **(B)** Diagnostic bone marrow flow cytometry demonstrating coexistence of an abnormal immature myeloid/blast population together with a CD5-negative/CD10-negative kappa-restricted mature B-cell population and a small clonal plasma-cell population sharing the same light-chain restriction. **(C)** Uniform manifold approximation and projection (UMAP) of all profiled bone marrow cells showing the major compartments, including AML-related myeloid cells, a WM-related B/plasma-cell compartment, T/NK cells, monocyte/niche cells, and erythroid/megakaryocytic cells. **(D)** Lineage-oriented dot plot showing representative marker expression across the major compartments; dot size indicates the fraction of cells expressing each marker, and color indicates scaled average expression. These clinicopathologic and single-cell findings are consistent with a collision marrow composed of AML with myelomonocytic features and a WM-related lymphoplasmacytic compartment.

At presentation, initial laboratory evaluation showed a white blood cell count of 6.2 × 10^9^/L, hemoglobin of 110 g/L, and platelet count of 7 × 10^9^/L, with 44% circulating blasts on the peripheral blood smear. Serum IgM was 25.8 g/L, with an IgM-kappa monoclonal protein by immunofixation. Bone marrow biopsy documented AML and a lymphoplasmacytic component, although the percentage of lymphoplasmacytic infiltration was not quantitatively reported; no definite multilineage dysplasia was identified on the available aspirate smear. Orthogonal molecular testing detected *MYD88* L265P, providing additional support for the WM/LPL-related nature of the kappa-restricted B/plasma-cell compartment. These baseline and longitudinal findings are summarized in **Figure 1A**, and representative diagnostic flow-cytometric findings are shown in **Figure 1B** and **Supplementary Figure 1**. Conventional cytogenetics was reported as normal but was based on limited evaluable metaphases. Comprehensive myeloid next-generation sequencing (NGS) was not performed because it was not reimbursed and the patient declined self-pay testing. *NPM1* and *FLT3-ITD/TKD* status were unavailable, and molecular AML subclassification and European LeukemiaNet (ELN) risk assignment therefore could not be completed. These clinicopathologic data established the coexistence of an AML compartment and a flow-validated, kappa-restricted WM-related B/plasma-cell compartment in the diagnostic marrow. Single-cell RNA sequencing of the untreated bone marrow resolved a complex cellular ecosystem, including malignant, lymphoplasmacytic, immune, and residual hematopoietic compartments.

Unsupervised clustering and lineage-oriented annotation identified a dominant AML-related myeloid compartment, a discrete WM-related B/plasma-cell compartment, T/NK cells, microenvironment monocytes, and erythroid/megakaryocytic populations (**Figure 1C**). The global UMAP therefore supported a collision marrow architecture in which myeloid malignant cells coexisted with a separate B/plasma-cell-associated disease component within the same specimen. A minor erythroid/megakaryocytic compartment was also identified, likely representing residual hematopoietic elements, and was not pursued in downstream disease-focused analyses.

This global architecture was further corroborated by lineage-marker expression across the major compartments (**Figure 1D**). AML-associated regions were characterized by immature and myeloid markers, including *CD34, KIT, SPINK2, MPO*, and *LYZ*, whereas the WM-related compartment was enriched for B-cell and plasmacytic markers, including *CD19, MS4A1, CD79A, IGHM, JCHAIN, MZB1, XBP1*, and *SDC1*. In parallel, the T/NK compartment showed expected lymphoid features such as *CD3D, IL7R, CD8A*, and *NKG7*. The marker profiles confirmed the separation of AML-related myeloid cells, WM-related B/plasma cells, and immune compartments within the same marrow ecosystem.

### 3.2 CopyKAT delineates a CNV-enriched AML compartment with hierarchical myeloid transcriptional states

**Figure 2:**
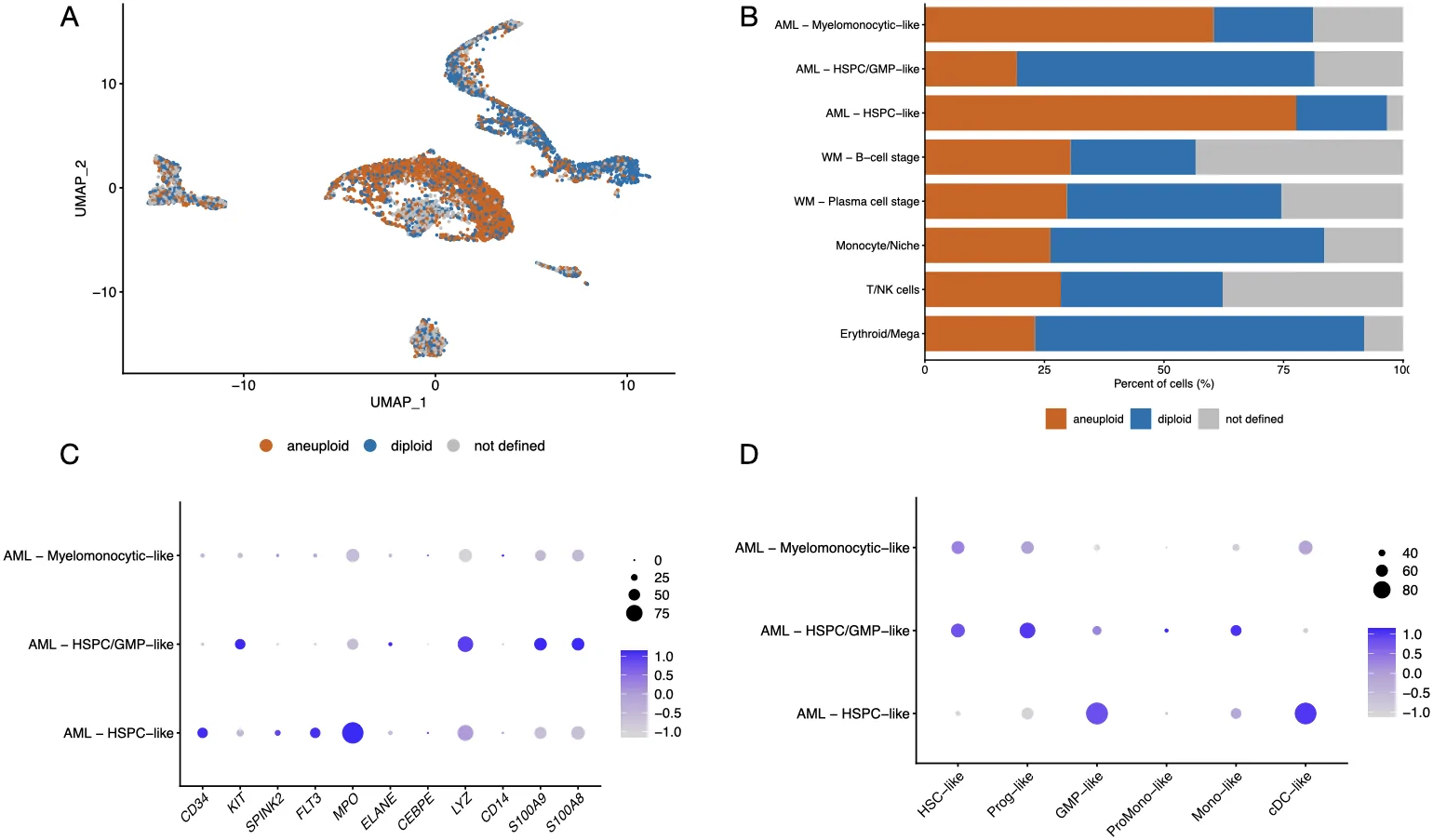
Copy-number inference localizes the dominant CNV-bearing population to the AML compartment and supports hierarchical myeloid organization. **(A)** Global UMAP overlaid with CopyKAT assignments, showing concentration of aneuploid cells within the AML-associated region. **(B)** Compartment-level distribution of aneuploid, diploid, and undefined CopyKAT calls across the major marrow populations. **(C)** Dot plot of representative markers highlighting three AML transcriptional states: an immature/progenitor-like program corresponding to the AML-HSPC-like state, a granulocytic-primed program corresponding to the AML-HSPC/GMP-like state, and an inflammatory/myelomonocytic-like program corresponding to the AML-myelomonocytic-like state. **(D)** Reduced Van Galen framework scoring across AML-associated populations, showing concordant enrichment of HSC-like, progenitor-like, GMP-like, cDC-like, promonocyte-like, and monocyte-like programs. These analyses support a hierarchically organized AML compartment rather than a transcriptionally uniform blast population.

To further resolve the malignant architecture of the marrow, we performed single-cell copy-number inference using CopyKAT (11). Among 9,019 cells, 4,059 (45.0%) were classified as aneuploid, 3,110 (34.5%) as diploid, and 1,850 (20.5%) as undefined. On the whole-marrow UMAP, aneuploid cells were concentrated within the central AML-associated region, whereas the majority of peripheral immune-associated clusters were predominantly diploid or undefined, indicating that the major CNV-bearing compartment resided within the AML system (**Figure 2A**).

Compartment-level CopyKAT composition showed that AML-associated populations had the highest enrichment of aneuploid calls, whereas non-AML compartments were predominantly diploid or showed mixed assignments (**Figure 2B**). The WM-related B/plasma-cell compartment did not show comparable CNV enrichment. Thus, the transcriptome-derived CNV signal was largely confined to AML-associated regions; CopyKAT did not independently establish or exclude clonality in the WM-related compartment.

Within the AML compartment, transcriptional heterogeneity was substantial and was not consistent with a single homogeneous blast population. Instead, AML-associated cells spanned layered myeloid states (**Figure 2C**). One subset displayed an immature/progenitor-like program corresponding to the AML-HSPC-like state in **Figure 2C**, marked by *CD34, KIT, SPINK2*, and *FLT3*; a second, corresponding to the AML-HSPC/GMP-like state, showed granulocytic priming, with expression of *MPO*, *ELANE*, and *CEBPE*; and a third, corresponding to the AML-myelomonocytic-like state, exhibited inflammatory/myelomonocytic features characterized by *LYZ, CD14, S100A8*, and *S100A9*. This organization was corroborated by reduced-state annotation using a reduced Van Galen framework (6), which demonstrated concordant enrichment of HSC-like, progenitor-like, GMP-like, cDC-like, promonocyte-like, and monocyte-like signatures across AML-associated populations (**Figure 2D**). This layered architecture, particularly the inflammatory/myelomonocytic component, was broadly concordant with the clinical impression of AML showing myelomonocytic features. The AML compartment was therefore CNV-enriched and organized across hierarchical myeloid transcriptional states.

We next examined BCL2-family expression across the diagnostic AML transcriptional states as a descriptive assessment of baseline apoptotic-regulator heterogeneity. *MCL1* was detectable across AML states and showed relatively higher scaled average expression in the AML-myelomonocytic-like state, whereas *BCL2* expression was comparatively higher in the AML-HSPC/GMP-like state and low in the myelomonocytic-like state. *BCL2L1* showed minimal expression, while *BCL2A1* was detected in a small fraction of cells, mainly within the myelomonocytic-like state (**Supplementary Figure 5**). These within-sample patterns were not tested as predictors of clinical response or resistance.

### 3.3 WM-related cells form a *MYD88*-supported, flow-validated compartment comprising coexisting B-cell-like and plasma-cell-like states with heterogeneous CNV assignment

**Figure 3:**
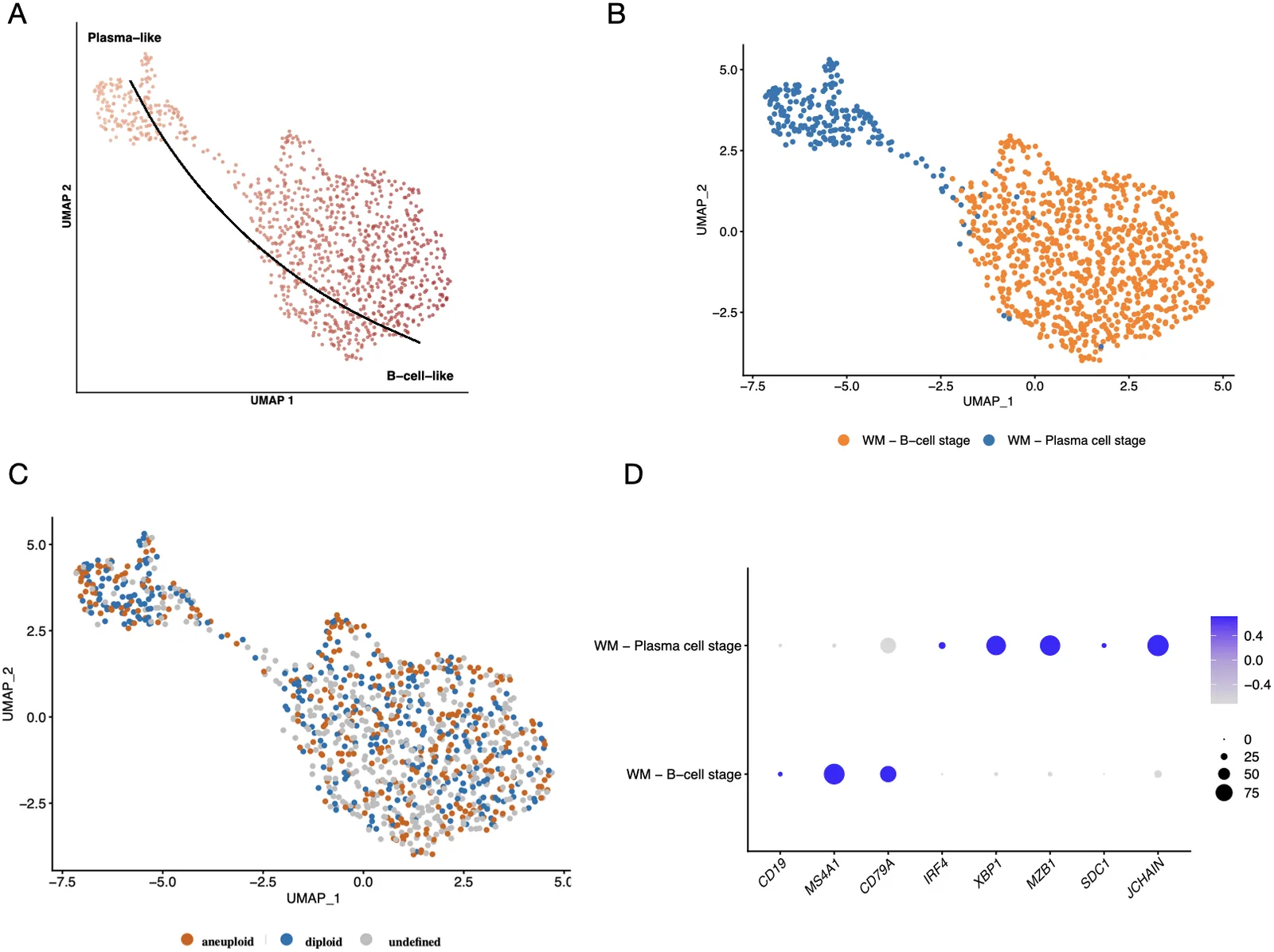
WM-related cells comprise coexisting B-cell-like and plasma-cell-like transcriptional states with limited CNV resolution by transcriptome-based inference. **(A)** UMAP of the WM-related compartment showing a connected state map comprising B-cell-like and plasma-cell-like regions. **(B)** State annotation across the WM-related phenotypic spectrum, highlighting B-cell-like and plasma-cell-like regions. **(C)** Projection of CopyKAT assignments onto the WM-related state map, showing interspersed aneuploid, diploid, and undefined cells rather than a discrete CNV-defined region. **(D)** Dot plot of representative state-associated markers, with B-cell-like cells enriched for *CD19, MS4A1*, and *CD79A* and plasma-cell-like cells enriched for *IRF4, XBP1, MZB1, SDC1*, and *JCHAIN.* In conjunction with flow cytometry, monoclonal IgM, and *MYD88* L265P testing, these findings support a WM-related lymphoplasmacytic process with coexisting B-cell-like and plasma-cell-like states.

The WM-related compartment formed a connected state map containing B-cell-like and plasma-cell-like regions (**Figures 3A, B**). These regions were interpreted as coexisting transcriptional states without a directional lineage relationship. B-cell-like regions preferentially expressed *CD19*, *MS4A1*, and *CD79A*, whereas plasma-cell-like regions showed higher *IRF4, XBP1, MZB1, SDC1*, and *JCHAIN* expression (**Figure 3D**).

When CopyKAT calls were projected onto this WM-related state map, aneuploid, diploid, and undefined cells were interspersed rather than segregating into a discrete CNV-defined cluster (**Figure 3C**). Thus, unlike the AML compartment, the WM-related population was not captured as a uniformly CNV-enriched malignant block by transcriptome-based copy-number inference. Its clonal nature was instead supported by marrow flow cytometry demonstrating a kappa-restricted mature B-cell population together with a small clonal plasma-cell population sharing the same restriction (**Figure 1B**), monoclonal IgM, and MYD88 L265P (**Figure 1A**). *MYD88* L265P was treated as supportive rather than diagnostic in isolation. After treatment, the abnormal mature B-cell population decreased substantially but remained detectable at a low level (0.1%) despite marked reduction of the AML burden (**Figure 1A**). These findings describe incomplete early clearance of the WM-related compartment without implying synchronous response or a molecularly resolved second clone.

Because broad separation of the major compartments was expected to be driven largely by cell identity, we next examined selected compartment-biased and broadly expressed transcriptional programs beyond lineage assignment. The AML-related and monocyte/niche compartments showed overlapping myeloid and inflammatory programs, including LYZ, S100A8, S100A9, FCN1, CST3, TYROBP, and CTSS. The WM-related compartment showed B/plasma-cell programs, including JCHAIN, MZB1, XBP1, IRF4, CD79A, MS4A1, and IGHM. Broadly expressed immune/niche-associated genes, including HLA-A, HLA-B, HLA-C, HLA-E, CD74, LGALS9, CD44, PTPRC, CXCR4, B2M, IFITM2, and IFITM3, were detected across multiple compartments, supporting a shared immune context within the same marrow specimen without implying collision-specific biomarkers (**Supplementary Figure 6A**).

Reference-guided signature scoring showed partial alignment of AML states with published programs: Mono_like scores were higher in the AML-myelomonocytic-like state, while HSC/GMP-related scores were distributed across immature/progenitor-like states. MBC_like and PC_like score distributions within the combined WM-related compartment provided descriptive reference context but were not formal between-state comparisons (**Supplementary Figure 6B**). These analyses were exploratory and were not designed to define formal molecular subtypes.

### 3.4 Exploratory immune-context analysis and early treatment kinetics

**Figure 4:**
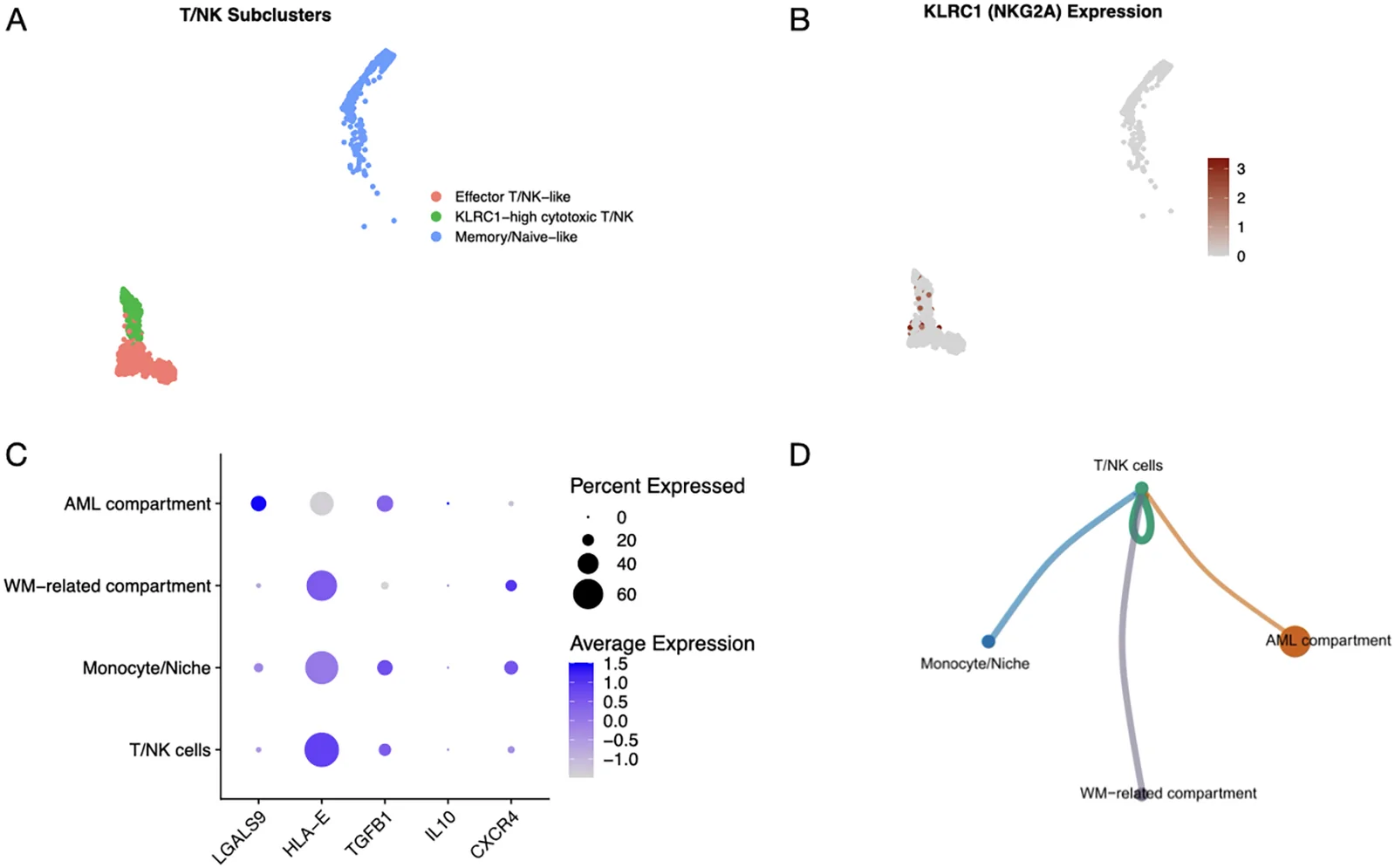
Exploratory immune-context analysis identifies a *KLRC1*-high cytotoxic T/NK subset and transcriptome-inferred T/NK-centered immunoregulatory signaling. UMAP of T/NK subclustering identifying memory/naive-like, effector-like, and *KLRC1*-high cytotoxic states. **(B)** Feature plot showing *KLRC1* (NKG2A) expression across the same T/NK UMAP, with enrichment in the *KLRC1*-high cytotoxic state. **(C)** Dot plot showing compartment-level expression of *LGALS9, HLA-E, TGFB1, IL10*, and *CXCR4* across AML, WM-related, monocyte/niche, and T/NK compartments; dot size indicates the fraction of cells expressing each gene, and color indicates scaled average expression. **(D)** CellChat network summary of T/NK-directed signaling within the MHC-I module, with the T/NK compartment as the principal predicted receiver; directed edges indicate inferred signaling from sender to receiver. These panels are descriptive and hypothesis-generating and do not demonstrate functional inhibition or validated cell-cell interactions.

To characterize the immune context of the collision marrow, we subclustered the T/NK-cell compartment and identified three major transcriptional states: a memory/naive-like population, an effector T/NK-like population, and a discrete *KLRC1*-high cytotoxic T/NK subset (**Figure 4A**). Feature mapping showed enrichment of *KLRC1*, which encodes *NKG2A*, within this subset (**Figure 4B**). These findings are compatible with an inhibitory-receptor-associated phenotype but do not establish functional inhibition.

Focused expression profiling showed partially overlapping but compartment-biased expression of *LGALS9, HLA-E, TGFB1, IL10*, and *CXCR4* across AML, WM-related, T/NK, and monocyte/niche compartments (**Figure 4C**). Complementary cell-cell communication analysis within the MHC-I module identified the T/NK compartment as the principal predicted receiver, with sender contributions from the T/NK autocrine loop, the WM-related, the monocyte/niche, and the AML compartment (**Figure 4D**). Because ligand-receptor inference from single-cell transcriptomes is probabilistic and method-dependent (13), these transcriptome-derived predictions are presented as hypotheses rather than as evidence of physical contact or functional signaling, and no inferred interaction was validated at the protein or functional level. Full CellChat outputs, including global interaction summaries (**Supplementary Figure 2**), *GALECTIN* pathway views (**Supplementary Figure 3**), and an overall T/NK-targeted chord summary complementary to the network shown in **Figure 4D** (**Supplementary Figure 4**), are presented in the **Supplementary Appendix**.

The distinction between the two coexisting disease compartments was further reflected in their longitudinal treatment responses (**Figure 1A**). After azacitidine plus venetoclax, marrow flow cytometry showed a marked reduction in the AML blast population from 35.9% at diagnosis to 0.2% in the early post-treatment sample. The WM-related mature B-cell population also decreased substantially but remained detectable at 0.1%, while monoclonal IgM declined more slowly and did not fully normalize during follow-up. A subsequent marrow examination demonstrated re-emergence of abnormal blasts (4.4%) together with persistent low-level kappa-restricted B cells (0.1%). The two marrow compartments therefore showed different early treatment kinetics, with deeper early marrow clearance of AML and incomplete hematologic and serologic resolution of the WM-related process.

## 4 Discussion

This study describes a collision marrow containing a dominant CNV-bearing AML compartment with hierarchical myeloid states and a separate flow-validated, kappa-restricted WM-related B/plasma-cell compartment with coexisting B-cell-like and plasma-cell-like states. Its contribution is descriptive and methodological rather than diagnostic or mechanistic: routine morphology and flow cytometry established lineage coexistence, whereas single-cell profiling localized the dominant CNV signal, resolved within-compartment state organization, described baseline BCL2-family heterogeneity, and nominated shared immune/niche programs. The study does not claim that single-cell sequencing is required to distinguish AML from a B-cell clone or that the observed programs are unique to collision marrow.

Within the AML compartment, CopyKAT localized the dominant CNV-bearing population to AML-associated regions, and marker-based state analysis distributed these cells across immature/progenitor-like, granulocytic-primed, and inflammatory/myelomonocytic-like programs. Annotation using a reduced Van Galen framework was concordant with this interpretation (6). The hierarchy is consistent with prior AML single-cell atlases; the present case adds only the observation that such state organization can be examined alongside a separate WM-related compartment in the same specimen.

The WM-related compartment is supported by concordant flow-cytometric, serologic, and molecular findings. Clinically and pathologically, WM is defined by monoclonal IgM together with marrow infiltration of clonal lymphoplasmacytic cells (1). In our case, the WM-related population was supported by marrow flow cytometry demonstrating a kappa-restricted CD5-negative/CD10-negative mature B-cell population together with a small clonal plasma-cell population sharing the same restriction, alongside serum evidence of monoclonal IgM including immunofixation electrophoresis and *MYD88* L265P mutation.

On single-cell analysis, this compartment contained coexisting B-cell-like and plasma-cell-like transcriptional states. This pattern reflects shared transcriptional-state organization within a WM-related lymphoplasmacytic compartment but does not indicate local derivation of plasma-cell-like cells from the observed marrow B-cell-like population. The WM clone is widely considered to be a post-germinal-center, somatically hypermutated IgM^+^ memory B cell (1). Therefore, the plasma-cell-like states detected in the marrow may have been generated at other anatomical sites and subsequently localized to the marrow. Their anatomical origin, temporal order, and local differentiation direction cannot be determined from single-cell transcriptomic data alone. This coexisting-state interpretation is consistent with recent single-cell and multiomic studies describing memory-B-cell-like and plasma-cell-like molecular programs in WM (10, 14). Mixed CopyKAT assignments and the absence of matched DNA sequencing further preclude presenting this population as a fully molecularly resolved second malignant clone.

The two compartments showed different early treatment kinetics. In our patient, therapy was designed primarily around AML using azacitidine plus venetoclax; the AML blast burden fell sharply, and the WM-related B-cell compartment also decreased substantially by flow cytometry. However, low-level kappa-restricted cells remained detectable, serum IgM did not normalize, and immunofixation remained positive for IgM-kappa. This discordance should not be taken as proof of WM-specific venetoclax resistance. The regimen was AML-directed, and WM response assessment integrates both marrow disease and serum IgM, which may improve on different timescales (15, 16). A previous case similarly reported that azacitidine plus venetoclax can induce clinical responses in a patient with concomitant WM and a myeloid neoplasm, although the depth and tempo of clearance across compartments may differ (17).

At the molecular level, our data cannot define venetoclax resistance transcripts, because single-cell profiling was performed only at diagnosis. The pretreatment AML compartment showed descriptive heterogeneity in BCL2-family transcripts, including relatively higher MCL1 and lower BCL2 expression in the AML-myelomonocytic-like state (**Supplementary Figure 5**). This pattern is biologically compatible with prior observations linking monocytic differentiation to reduced BCL2 dependence and lower venetoclax sensitivity (18, 19), although monocytic differentiation alone is not a universal predictor of venetoclax response (20). These baseline observations cannot account for the later AML re-expansion, which was not profiled by single-cell sequencing, and no causal or predictive association with treatment resistance is claimed.

The immune analysis was likewise exploratory. T/NK subclustering suggested that the marrow was not uniformly dominated by terminally exhausted-like states, but instead contained an effector-dominant compartment together with a smaller *KLRC1*-high cytotoxic T/NK state. Focused expression profiling and CellChat nominated MHC-I signaling and GALECTIN-related patterns involving multiple marrow compartments. During the available 5-month follow-up, both disease-related compartments initially decreased, followed by AML re-expansion, persistent low-level kappa-restricted B cells, and sustained IgM-kappa serologic abnormality. These longitudinal observations document dissociated kinetics but do not identify their mechanism.

Broadly similar immune features have been reported in WM single-cell studies, including altered cytokine-associated communication and reduced T/NK interferon responsiveness (9, 10). The present data, however, cannot establish whether these patterns are shared, disease-specific, or related to the coexistence of two neoplasms. Cell-cell communication inference from single-cell transcriptomes remains probabilistic and depends on the analytical method and the ligand-receptor resource (12, 13). No inferred interaction was validated by flow cytometry, protein-based spatial assays, or perturbation experiments. We therefore interpret the network results only as hypotheses for future validation, not as functional proof of specific signaling dependencies.

Additional limitations include the single-case design, absence of patient-matched normal or single-neoplasm controls, absence of matched DNA sequencing and comprehensive myeloid NGS, limited evaluable metaphases, a lack of post-treatment single-cell profiling, and a lack of spatial or functional validation. Although no definite multilineage dysplasia was identified on the available aspirate smear, incomplete genetic assessment prevents exclusion of myelodysplasia-related or antecedent clonal biology. This is relevant because an unrecognized antecedent clone may itself have altered the marrow microenvironment, and malignant clones and the marrow niche are known to shape one another reciprocally (21); the immune and niche-associated programs described here therefore cannot be attributed specifically to the coexistence of the two neoplasms. Individual cells were not treated as independent biological replicates, and no collision-specific signature can be distinguished from inter-individual variability in this n = 1 study; raw integration with external cohorts is further confounded by batch effects (22).

Future studies should use harmonized multi-patient cohorts with matched AML-only, WM/LPL-only, and non-malignant marrow controls, longitudinal sampling before and after treatment, and paired genomic profiling. Spatial transcriptomics or spatial proteomics could then preserve tissue architecture and test the physical proximity and interaction patterns among coexisting malignant clones, immune cells, and niche elements.

## 5 Conclusion

In this single diagnostic marrow, single-cell RNA sequencing complemented standard diagnostics by resolving AML state architecture and a separate WM-related B/plasma-cell state spectrum while nominating broadly shared immune programs. It did not establish collision-specific biomarkers, treatment-resistance mechanisms, lineage directionality, or functional cell-cell interactions. The case supports compartment-aware monitoring and provides a rationale for matched longitudinal, genomic, and spatial studies of composite marrow malignancies.

## Data Availability

The raw and processed single-cell RNA-sequencing data generated in this study have been deposited in the NCBI Gene Expression Omnibus (GEO) under accession number GSE343373 and will be publicly available upon release.
