# Supplementary material for: Single-cell dissection of a collision marrow with coexisting acute myeloid leukemia and a Waldenström-related lymphoplasmacytic compartment
Source: Front Oncol. 2026 Sep 11;16:1844537. doi: 10.3389/fonc.2026.1844537 (PMC13612327; doi:10.3389/fonc.2026.1844537)
Supplement: Supplementary file 1 [file Table1.docx]

**Supplementary Figure S1. Longitudinal flow-cytometric follow-up of the coexisting marrow compartments.**

(A) Representative bone marrow flow-cytometry plots from the early post-treatment marrow reassessment (2 months after diagnosis). From left to right, plots show global gating on CD45 versus side scatter, the residual blast gate in relation to CD34 and CD13, identification of the mature B-cell compartment by CD19 and CD20, and kappa/lambda light-chain assessment of the gated B-cell population. Abnormal blasts were markedly reduced at this time point, whereas a small residual kappa-restricted mature B-cell population remained detectable.

(B) Representative bone marrow flow-cytometry plots from the later marrow reassessment (5 months after diagnosis). The same gating strategy is shown. Compared with the early reassessment, abnormal blasts had re-emerged, while a low-level kappa-restricted mature B-cell population persisted. These longitudinal flow-cytometric findings complement the summary shown in Figure 1A and illustrate different early treatment kinetics across the AML and WM-related marrow compartments.

**Supplementary Figure S2. Global CellChat interaction summaries across major marrow compartments.**

(A) Global CellChat network showing the number of inferred interactions among the major marrow compartments, including the AML compartment, WM-related compartment, T/NK cells, and monocyte/niche cells.

(B) Global CellChat network showing the corresponding interaction strength (communication probability) across the same compartments.

**Supplementary Figure S3. GALECTIN pathway views across major marrow compartments.**

(A) CellChat-inferred GALECTIN signaling network across the AML compartment, WM-related compartment, T/NK cells, and monocyte/niche cells.

(B) T/NK-directed view of the inferred GALECTIN pathway, highlighting communication edges converging on the T/NK compartment.

(C) Dot plot showing compartment-level expression of selected GALECTIN pathway-related genes, including LGALS9, PTPRC, and CD44. Dot size indicates the fraction of expressing cells, and color indicates scaled average expression.

(D) Relative contribution of inferred ligand-receptor pairs within the GALECTIN pathway, with LGALS9–CD45 contributing more strongly than LGALS9–CD44 in this analysis.

**Supplementary Figure S4. Overall chord summary of T/NK-associated communication across major marrow compartments.**

Chord diagram summarizing the aggregate communication landscape with emphasis on T/NK-associated interactions across the major marrow compartments, including the AML compartment, WM-related compartment, monocyte/niche cells, and the T/NK autocrine loop. Ribbon width is proportional to the inferred aggregate communication probability. This panel provides an overall pathway-agnostic summary complementary to the MHC-I-centered network shown in Figure 4D and illustrates that multiple marrow compartments contribute to the transcriptome-inferred immunoregulatory environment surrounding T/NK cells.

**Supplementary Figure S5. Baseline BCL2-family expression across diagnostic AML transcriptional states.**

Dot plot showing expression of BCL2-family regulators across diagnostic AML transcriptional states. Dot size indicates the fraction of cells expressing each gene, and color indicates scaled average expression. Expression of apoptosis-regulatory transcripts was sparse, as commonly observed in 3’ scRNA-seq data; therefore, percent expressed and average expression were interpreted together. The comparison is descriptive; individual cells were not treated as independent biological replicates, and no inferential statistical test or prediction of treatment response was performed.

**Supplementary Figure S6. Compartment-biased and broadly expressed transcriptional programs.**

(A) Dot plot showing selected transcriptional programs across AML-related cells, WM-related cells, monocyte/niche cells, and T/NK cells. Genes are grouped into myeloid/inflammatory genes, B/plasma-cell genes, and broadly expressed immune/niche-associated genes, separated by dashed vertical lines. Dot size indicates the fraction of cells expressing each gene, and color indicates scaled average expression.

(B) Reference-guided scoring of AML and WM-related transcriptional programs. Violin plots show HSC-like, GMP-like, and monocyte-like scores across AML transcriptional states and MBC-like and PC-like score distributions within the combined WM-related compartment. The WM-related violins are not formal between-state comparisons. These n=1 analyses are descriptive and were not designed to define collision-specific biomarkers, formal molecular subtypes, or treatment-response associations.
